# Genome sequence of the type strain CLIB 1764^T^ (= CBS 14374^T^) of the yeast species *Kazachstania saulgeensis* isolated from French organic sourdough

**DOI:** 10.1016/j.gdata.2017.07.003

**Published:** 2017-07-04

**Authors:** Véronique Sarilar, Lieven Sterck, Saki Matsumoto, Noémie Jacques, Cécile Neuvéglise, Colin R. Tinsley, Delphine Sicard, Serge Casaregola

**Affiliations:** a Micalis Institute, INRA, AgroParisTech, CIRM-Levures, Université Paris-Saclay, 78350 Jouy-en-Josas, France; b Ghent University, Department of Plant Biotechnology and Bioinformatics, Technologiepark 927, 9052 Ghent, Belgium; c VIB Center for Plant Systems Biology, 9052 Ghent, Belgium; d Micalis Institute, INRA, AgroParisTech, Université Paris-Saclay, 78350 Jouy-en-Josas, France; e Sciences pour l'œnologie, INRA, Supagro, Université de Montpellier, 34060 Montpellier, France

**Keywords:** Saccharomycotina, Yeast, Sourdough, *Kazachstania*, Genome

## Abstract

*Kazachstania saulgeensis* is a recently described species isolated from French organic sourdough. Here, we report the high quality genome sequence of a monosporic segregant of the type strain of this species, CLIB 1764^T^ (= CBS 14374^T^). The genome has a total length of 12.9 Mb and contains 5326 putative protein-coding genes, excluding pseudogenes and transposons. The nucleotide sequences were deposited into the European Nucleotide Archive under the genome assembly accession numbers FXLY01000001–FXLY01000017.

**Table:** 

Specifications	
Organism/strain	*Kazachstania saulgeensis* strain CLIB 1764^T^
Sex	N/A
Sequencer or array type	Illumina HiSeq 2500, mate pair libraries
Data format	Processed data: genome assembly and annotated embl files
Experimental factors	N/A
Experimental features	Genomic DNA extracted from pure yeast
Consent	N/A
Sample source location	Sourdough samples obtained from baker Michel Perrin at *Ferme des plants*, Saulgé, France (46° 20′ 27.08″ N 0° 53′ 12.21″ E)

## 1 Direct link to deposited data

https://www.ncbi.nlm.nih.gov/bioproject/PRJEB20516.

## 2 Introduction

The role of yeasts in bread making involves leavening the dough by fermenting carbon sources present in flour and producing aroma. In addition to the baker's yeast *Saccharomyces cerevisiae*, a number of other yeast species can be found in dough, in particular *Torulaspora delbrueckii*, *Wickerhamomyces anomalus* and *Pichia kudriavzevii* along with *s*everal members of the genus *Kazachstania*, such as *Candida humilis* (syn. *Candida milleri*, now *Kazachstania humilis*), *Kazachstania exigua*, less frequently *Kazachstania bulderi* and *Kazachstania unispora* [1], [2]. A recent analysis of French organic sourdough revealed the presence a novel species, *Kazachstania saulgeensis* [2], [3]. Here we report a high quality draft of the genome sequence of a monosporic segregant of the type strain of this species. The availability of the genome of *K. saulgeensis* will facilitate studies on the role of nonconventional yeasts in dough and the search for alternative baker's yeasts with interesting properties such as novel natural aromas.

## 3 Experimental design, materials and methods, results

Spore isolation from strain CLIB 1764^T^ grown on malt agar was performed as described in [4]. DNA from a single spore grown on YPD medium was prepared as previously described [4]. Preparation of two mate-pair libraries from the purified DNA and sequencing (Illumina HiSeq 2500 platform) was performed by BGI Genomics, Shenzhen, China. Two mate-pair libraries of 6-kbp insert size were sequenced, generating 6,055,467 read pairs of 100 bp and 5,496,657 read pairs of 125 bp. After trimming according to quality criteria with Trimmomatic [5], 21,095,636 reads were retained, leading to an apparent 190-fold coverage. The reads were assembled using Platanus, v1.2.1 [6] with default parameters. GapCloser v1.12 [7] was used to fill gaps where possible. The resulting assembly consisted of 3748 scaffolds with a maximum length of 2.96 Mb and with an N50 length of 1.37 Mb. The cumulative size was 13.99 Mb. The rDNA unit was assembled separately and manually integrated between the two scaffolds identified as being next to rDNA after mate-pair read mapping using BWA [8]. The resulting scaffold containing the rRNA locus was 0.89 Mb in size.

Annotation was performed on the 17 scaffolds larger than 10 kb (cumulative size of 12,935,755 bp, 32.5% GC content), whose size varied from 17.3 kb to 2.95 Mb (Table 1).

**Table 1:** Genome statistics for the strain CLIB 1764^T^.

Attribute	CLIB 1764^T^
Genome size (bp)	12,935,755
Scaffolds > 10 kb	17
N50	1.37 Mb
G + C content	33%
Protein coding genes	5326
Pseudogenes	38
tRNA genes	197
LTR-retrotransposons (including pseudogenes)	15
Solo Long Terminal Repeats	278
DNA transposons (including. pseudogenes)	6

Based on the reference genomes of two related and well annotated, species belonging to the *Saccharomycetaceae*, *Saccharomyces cerevisiae* (http://www.yeastgenome.org/) and *Lachancea kluyveri* [9], a total of 5326 putative protein coding genes (CDS) and 38 pseudogenes were found using the Amadea Annotation transfer tool (Isoft, France). Functional annotation was performed based on protein similarity with *S. cerevisiae*. Coding sequences with no similarity to those in *S. cerevisiae* were annotated using the refseq and nr databases at NCBI. Further putative CDS were added after prediction of CDS longer than 150 aa with ORF Finder (http://www.ncbi.nlm.nih.gov/orffinder/) and blast analysis against the NCBI non redundant database, to yield a total of 5326 CDS (Table 1). Some of the gene models were manually curated on the ORCAE platform (http://bioinformatics.psb.ugent.be/orcae/; [10]) and visualized on GenomeView (http://genomeview.org; [11]). Interestingly, an arginase, whose gene had no equivalent in Saccharomycotina yeasts, but which presented strong sequence similarities with those of *Penicillium* is very likely the result of a horizontal gene transfer event.

One entire and one partial *Ty3*/gypsy retrotransposon were identified, together with 13 *Ty*-like pseudogenes. A total of 278 Long Terminal Repeats from retrotransposons were identified, belonging to at least 10 subfamilies. One of these subfamilies displays an unusual size of 714 bp, reminiscent of the long LTR found in *Kazachstania exigua* [12]. Members of two families of hAT DNA transposons, *Roamer* and *Rover* [13], [14], [15] with four and two elements respectively, were also identified; all were pseudogenes. A total of 197 tRNA were identified, using tRNAscan-SE v1.3.1 [16] (Table 1).

**Fig. 1:**
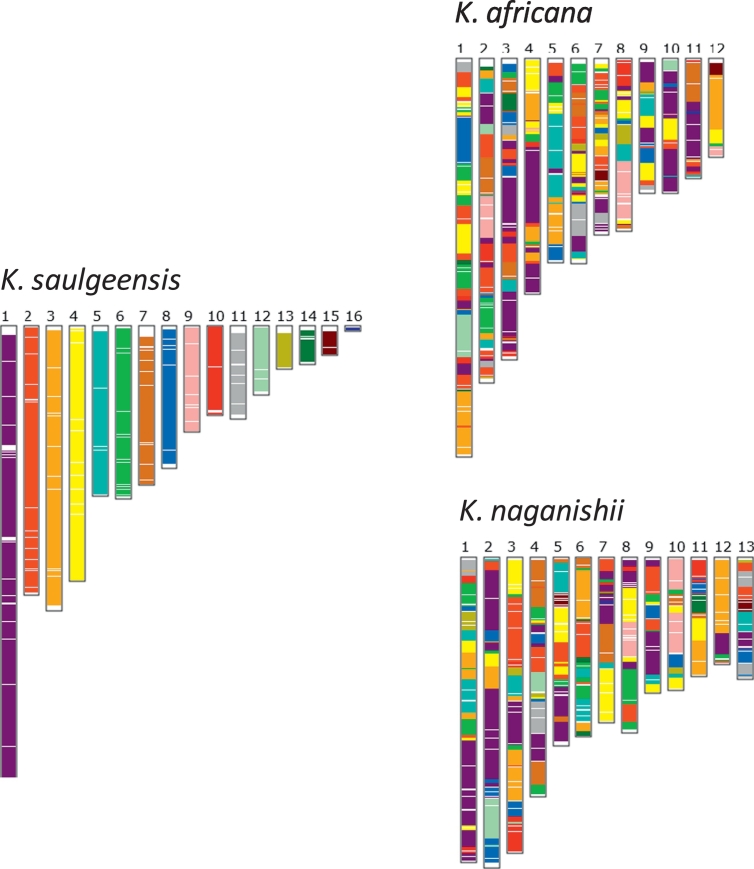
Synteny blocks between the genomes of *K. saulgeensis* and two other *Kazachstania* species. Orthology relationships between genes from *K. africana*, *K. naganishii* and *K. saulgensis* were defined on the basis of bidirectional hits in a blastp comparison (reciprocal best hits) computed by SynChro [18]. The color attributed to the genes of a given *K. saulgeensis* scaffold is conserved for their counterparts in *K. africana* and *K. naganishii*.

We used the available genome of the type strain of two *Kazachstania* species, *Kazachstania africana* and *Kazachstania naganishii*, to investigate chromosome colinearity between *K. saulgeensis* and these species [17]. We examined the synteny based on the presence and order of orthologous genes using SynChro [18], with Delta = 4 to minimize artifactual synteny breaks. This showed that rearrangements that have occurred since the last common ancestor of *K. saulgeensis*, *K. africana* and *K. naganishii* are numerous and affect each scaffold equally (Fig. 1).

## 4 Nucleotide accession number

The genome sequences generated in this study are available from the European Nucleotide Archive under the genome assembly accession number GCA_900180425 and the scaffold accession range FXLY01000001–FXLY01000017. The genome can be browsed and searched at http://bioinformatics.psb.ugent.be/orcae/overview/Kasa.

## Conflict of interest statement

The authors declare no conflict of interest.
